# Acute Neuromuscular Responses to Combined Electrical Stimulation and Blood Flow Restriction: Effects on Quadriceps Torque, Fatigue, and Pain

**DOI:** 10.7759/cureus.95026

**Published:** 2025-10-21

**Authors:** Salvador Santiago-Pescador, Susana López-Ortiz, Carlos Baladrón, Simone Lista, Enzo Emanuele, Piercarlo Minoretti, José Pinto-Fraga, Alejandro Santos-Lozano, Juan Martín-Hernández

**Affiliations:** 1 i+HeALTH Strategic Research Group, Department of Health Sciences, Miguel de Cervantes European University, Valladolid, ESP; 2 Cardiology Department, Hospital Clínico Universitario de Valladolid, Valladolid, ESP; 3 Scientific Directorate, 2E Science, Robbio, ITA; 4 General Direction, Studio Minoretti, Oggiono, ITA

**Keywords:** blood flow restriction, muscle fatigue, muscle torque, neuromuscular electrical stimulation, pain perception

## Abstract

Background and objective

Neuromuscular electrical stimulation (NMES) combined with blood flow restriction (BFR) offers a promising therapeutic strategy for enhancing muscle strength in clinical populations who are unable to engage in high-intensity voluntary exercise. However, the optimal combination of NMES intensity and BFR pressure remains unclear, especially in terms of achieving an effective therapeutic outcome while maintaining patient tolerability. In this study, we sought to investigate how varying NMES intensities (medium-to-high) combined with different BFR pressures may affect quadriceps muscle torque generation, fatigue development, and pain perception.

Methods

Sixteen healthy individuals (10 females and six males; mean age: 19.3 ± 2.9 years) underwent six randomized experimental conditions combining two NMES intensities (50% and 75% of maximum tolerable intensity) with three different BFR pressures: unrestricted flow (0 mmHg), moderate restriction (50 mmHg), and severe restriction (250 mmHg). Each condition consisted of one set of five isometric contractions. Outcome measures included evoked torque (assessed via isokinetic dynamometry), fatigue slope (calculated through linear regression analysis), and pain perception (evaluated using a visual analog scale (VAS).

Results

Evoked torque significantly decreased during the fourth and fifth repetitions under severe BFR conditions relative to initial repetitions (p = 0.016). The rate of fatigue development, as measured by slope analysis, was significantly accelerated in all BFR conditions compared to unrestricted circulation (p = 0.013). Pain scores were significantly higher under severe BFR pressure, with a dose-dependent relationship between NMES intensity and discomfort, where 75% of maximum tolerable intensity elicited significantly greater pain than 50% intensity (p = 0.001).

Conclusions

The combination of moderate-to-high NMES intensity with severe BFR pressure accelerates muscular fatigue while increasing pain perception. For clinical implementation, we recommend initiating protocols with 50% maximum tolerable NMES intensity combined with moderate BFR pressure (50 mmHg) to optimize therapeutic benefit while maintaining patient tolerability. Progression to higher intensities should be personalized based on the patient's response and level of adherence.

## Introduction

Neuromuscular electrical stimulation (NMES) combined with blood flow restriction (BFR) has emerged as a promising therapeutic modality for increasing muscle mass in clinical populations unable to perform conventional high-intensity exercise [1,2]. The effectiveness of NMES interventions typically depends on several factors, including anatomical considerations (notably motor nerve branching patterns), individual pain tolerance levels, and the precise selection of stimulation parameters [3]. Optimal force production can be achieved through biphasic rectangular pulses of 100-400 µs delivered at tetanic frequencies (50-100 Hz) at the subject’s maximum tolerable intensity, as this configuration maximizes motor unit recruitment through enhanced penetration of deeper muscle fibers [4]. Despite these optimization strategies, conventional NMES protocols may typically achieve only 40-60% of maximal voluntary contraction (MVC) [3], which may fall below the threshold required for substantial muscle hypertrophy or strength adaptations. This limitation primarily stems from the considerable discomfort generated by intense cutaneous and intramuscular electrical stimulation, which prevents subjects from tolerating currents sufficient to achieve full muscle activation [4].

In parallel, BFR training has gained recognition as an effective method for enhancing muscular adaptations during low-intensity resistance exercise [5], with research indicating results comparable to those achieved through traditional high-intensity resistance training [6]. The mechanistic basis for these adaptations involves significant upregulation of molecular pathways governing muscle hypertrophy [6]. However, widespread clinical adoption remains challenging, as higher BFR pressures, especially those nearing 60-80% of arterial occlusion pressure (AOP), can result in significant discomfort that often surpasses patients’ tolerance levels [7].

Recent investigations have demonstrated that combining low-intensity, low-frequency NMES with moderate BFR successfully promotes skeletal muscle hypertrophy and strength gains [1]. However, the concurrent application of electrical stimulation and external vascular pressure may ultimately create a unique sensory experience that can compromise patient adherence due to cumulative discomfort and accelerated fatigue [1,8]. Despite its clinical significance, the specific interactions between moderate-to-high NMES intensities and BFR on both perceptual and physiological responses are not yet well understood. To our knowledge, no previous investigations have systematically evaluated the effects of multiple submaximal NMES intensities when combined with standardized BFR pressures. This knowledge gap is particularly significant given that discomfort and fatigue represent primary determinants of treatment adherence in rehabilitation settings [9]. Hence, elucidating how medium-to-high NMES intensities interact with BFR to influence these parameters carries substantial implications for optimizing clinical protocols.

In light of this, the present investigation was designed to systematically evaluate how different BFR pressures combined with medium-to-high NMES intensities affect evoked torque production, fatigue progression, and pain perception in the quadriceps muscle. We hypothesized that both muscular fatigue and pain perception would demonstrate dose-dependent increases under conditions of elevated external pressure and higher NMES intensity, based on the principle that subjectively determined pain thresholds directly constrain achievable torque generation.

## Materials and methods

Sample size calculation

Sample size determination was informed by prior research [10] investigating reductions in evoked torque during combined BFR and NMES protocols. Based on an anticipated minimum difference of 47% in evoked torque between restricted and unrestricted conditions, a power analysis indicated that a sample of 16 participants would provide greater than 80% statistical power to detect significant effects at an alpha level of 0.05 (two-tailed). All calculations were performed using the G*Power software package (version 3.1.9.6).

Participants

Sixteen physically active young adults (10 females and six males; mean age: 19.3 ± 2.9 years; mean height: 170 ± 10.2 cm; mean weight: 62.1 ± 9.5 kg) voluntarily participated in the study. Eligibility criteria required participants to self-report as free from musculoskeletal, cardiovascular, metabolic, or pulmonary disorders and to have no prior experience with NMES training. Individuals were excluded if they presented with any musculoskeletal dysfunction that could potentially compromise performance testing. All enrolled participants completed the full experimental protocol. To minimize confounding variables, participants were instructed to abstain from stimulants and medications that could alter pain perception for at least 24 hours before each session. They were also required to avoid vigorous physical activity and to maintain consistent sleep patterns, dietary intake, and hydration habits throughout the study. The study adhered to the ethical standards of the Declaration of Helsinki and received approval from the Institutional Review Board of Miguel de Cervantes European University (protocol code CEI2017-001; approval date: April 27, 2018). Written informed consent was obtained from all participants following a detailed explanation of the study procedures.

Study design

**Figure 1:**
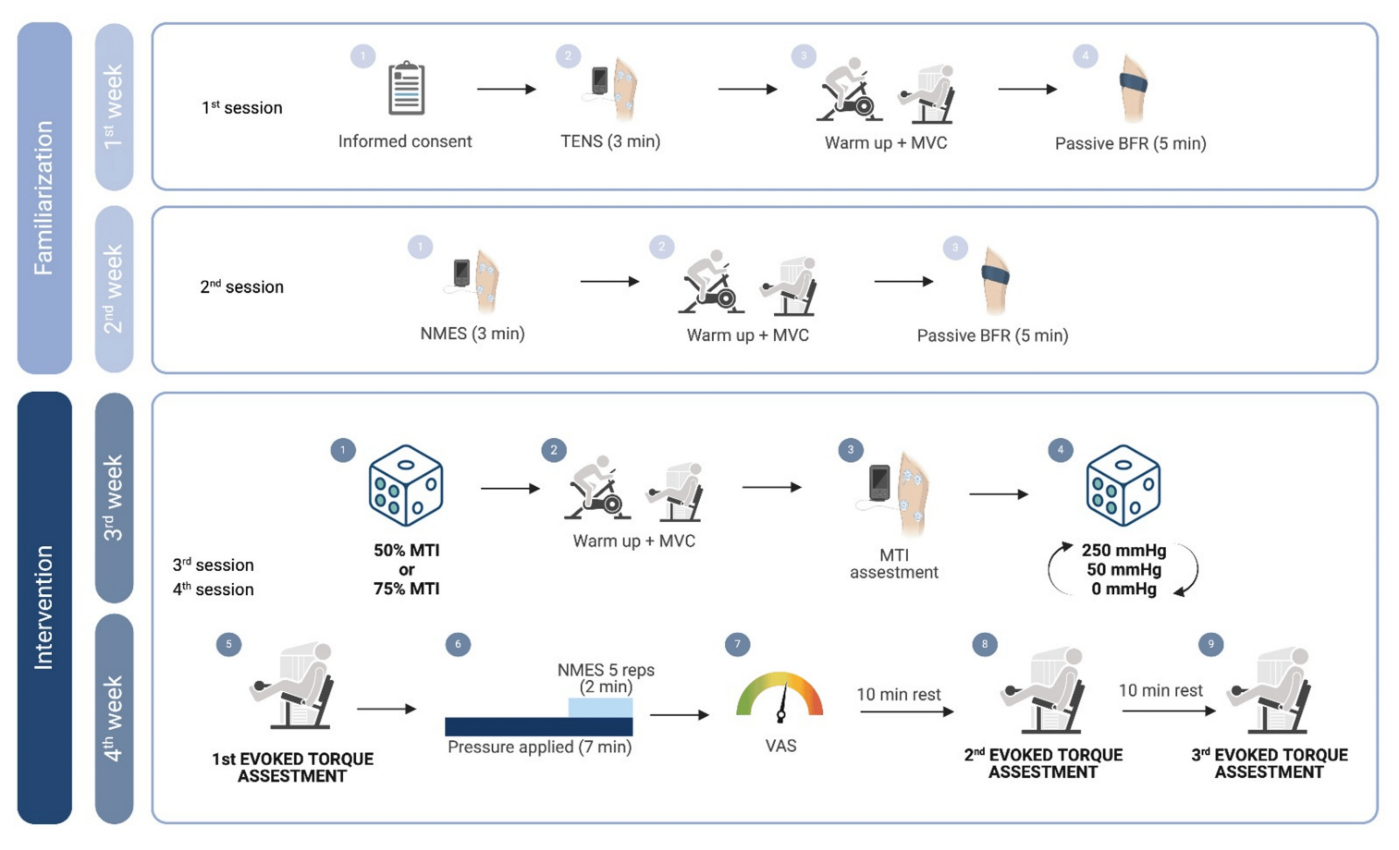
Experimental design summarizing the four-week study protocol The initial two weeks consisted of familiarization sessions introducing participants to TENS, NMES, and BFR procedures. The intervention phase (weeks 3-4) involved two sessions, each testing three randomized conditions combining different NMES intensities (50% or 75% MTI) with three BFR pressures (0, 50, or 250 mmHg). Each condition included five repetitions of electrically evoked contractions with 15-minute rest periods between conditions BFR: blood flow restriction; MTI: maximum tolerable intensity; MVC: maximal voluntary contraction; NMES: neuromuscular electrical stimulation; TENS: transcutaneous electrical nerve stimulation; VAS: visual analog scale Image created by the authors using BioRender

This randomized crossover study was conducted over four consecutive weeks. The initial two weeks consisted of familiarization sessions to ensure participant comfort with all procedures and equipment. The subsequent two-week intervention period involved exposure to six experimental conditions, with one testing session per week containing three conditions per session (Figure 1). Experimental conditions were established by combining two NMES intensities (50% and 75% of maximum tolerable intensity) with three BFR pressures: free circulation (0 mmHg, NMES), moderate restriction (50 mmHg, mild-BFR), and severe restriction (250 mmHg, sev-BFR). This resulted in six distinct conditions: 50NMES, 75NMES, 50mild-BFR, 75mild-BFR, 50sev-BFR, and 75sev-BFR. Participants were randomly assigned to their NMES intensity level, while the sequence of BFR pressure application was randomized within each session.

Familiarization protocol

The first familiarization session introduced participants to electrical stimulation through a three-minute transcutaneous electrical nerve stimulation (TENS) application to the quadriceps muscle. Following a standardized warm-up (five minutes of cycling at 70 watts, 60-70 rpm cadence), participants performed five unilateral isometric knee extensions and experienced five minutes of passive BFR at 250 mmHg to acclimate to occlusion sensations. The second session focused on familiarization with the specific NMES parameters used during testing, including practice reaching maximum tolerable intensity. Participants also completed MVC assessments and additional BFR exposure.

Intervention protocol

Each intervention session followed a standardized sequence. After the warm-up protocol, participants completed the MVC assessment followed by the determination of maximum tolerable NMES intensity. For MVC testing, participants performed three submaximal contractions (70%, 80%, and 90% of previously determined MVC) before executing five maximal isometric knee extensions (five-second contractions with 60-second rest intervals). Verbal encouragement to “push as hard and fast as possible” accompanied each maximal effort. Following a 10-minute recovery period, maximum tolerable NMES intensity was established by gradually increasing stimulation intensity from a baseline of 30% (determined during familiarization). Participants indicated when they reached their maximum tolerable threshold while maintaining muscle relaxation to avoid voluntary contribution.

Experimental conditions

A deflated BFR cuff was positioned on the dominant thigh five minutes before each condition. The randomized pressure was applied for seven minutes total, with NMES commencing after five minutes of restriction. Each condition involved five electrically evoked contractions (five-second stimulation, 20-second rest) for a total duration of 105 seconds. Participants were explicitly instructed to remain passive during stimulation to ensure torque resulted solely from electrical activation. A 15-minute rest period separated conditions to ensure complete recovery.

Neuromuscular electrical stimulation

Electrical stimulation was delivered using a programmable stimulator (Compex III, Medicompex Iberica, Barcelona, Spain) through three self-adhesive electrodes. One large negative electrode (10 × 5 cm, Easy Snap 2 × 4, Compex, DJO, Mouguerre, France) was positioned 10 cm below the inguinal crease, while two smaller positive electrodes (5 × 5 cm) were placed over the motor points of vastus medialis (WM) and vastus lateralis (VL) muscles. Motor point locations were determined according to manufacturer guidelines and marked with indelible ink to ensure consistent placement across sessions. Initial TENS parameters consisted of 100 Hz frequency and 50 µs pulse width at motor threshold intensity. The intervention NMES protocol utilized biphasic rectangular pulses with 380 µs pulse width delivered at 75 Hz.

Blood flow restriction protocol

An inelastic pneumatic cuff (140 mm width × 940 mm length; Riester Komprimeter, Jungingen, Germany) was applied to the proximal thigh. Three standardized pressures were selected to ensure consistent hemodynamic effects across participants: 0 mmHg (unrestricted flow), 50 mmHg (partial venous occlusion) [11], and 250 mmHg (complete vascular occlusion) [11,12]. These pressures were chosen to represent distinct physiological conditions rather than individualized percentages of arterial occlusion pressure.

Torque assessment

**Figure 2:**
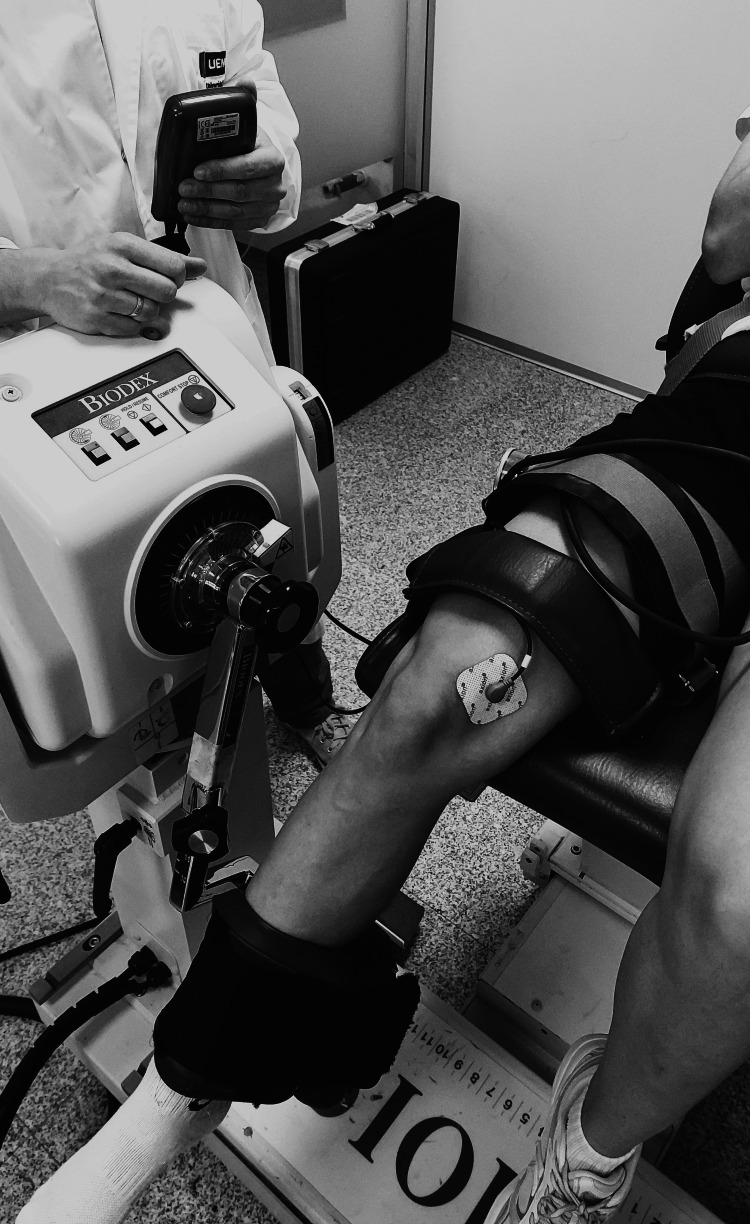
Experimental setup illustrating NMES combined with BFR during isometric knee extension Participants were seated on an isokinetic dynamometer with the dominant leg fixed at 60° knee and hip flexion. A pneumatic cuff was positioned on the proximal thigh to apply the assigned BFR pressure (0, 50, or 250 mmHg) during electrically evoked contractions. Self-adhesive electrodes were placed over the motor points of the quadriceps muscles to deliver the electrical stimulation. Participants remained passive throughout the stimulation to ensure that torque output reflected electrically induced activation only BFR: blood flow restriction; NMES: neuromuscular electrical stimulation

Isometric knee extension torque was measured using a calibrated isokinetic dynamometer (Biodex System IV, Biodex Medical Systems Inc., Shirley, NY). The testing position was standardized with 60° knee flexion and 60° hip flexion relative to anatomical position. The dynamometer axis was aligned with the knee joint axis of rotation, and participants were secured with straps across the thigh, pelvis, and chest to minimize extraneous movement (Figure 2). Evoked torque values were normalized to MVC to calculate relative peak strength.

Pain perception assessment

Pain perception was assessed immediately after each experimental condition using a visual analog scale (VAS). The VAS is a validated instrument comprising a 10-cm horizontal line, anchored at one end by “no pain” (0) and at the other by “worst possible pain” (10). This scale has demonstrated strong validity and reliability, exhibiting robust correlations with various measures of pain intensity [13].

Statistical analysis

Data normality was assessed using the Kolmogorov-Smirnov test. Unless otherwise specified, results are reported as mean ± standard deviation (SD) with accompanying 95% confidence intervals (CIs). Differences in evoked torque and relative peak strength were analyzed using a mixed repeated-measures analysis of variance (ANOVA), with repetition as the within-subjects factor and stimulation intensity (50% or 75%) and BFR pressure (0, 50, or 250 mmHg) as between-subjects factors. Bonferroni’s corrections were applied to all post hoc pairwise comparisons to control for family-wise error. Fatigue was quantified by calculating the slope of the linear regression between repetition number (independent variable) and evoked torque (dependent variable) [12]. Comparisons of fatigue slopes across experimental conditions were conducted using a one-way ANOVA, with Bonferroni’s post hoc tests for pairwise comparisons. Pain scores were analyzed using a mixed repeated-measures ANOVA, employing the same factorial design as the torque analysis. Bonferroni corrections were similarly applied to all post hoc comparisons. Analyses were performed using IBM SPSS Statistics, version 25.0 (IBM Corp., Armonk, NY), with all tests two-sided at a 5% level of significance.

## Results

Evoked torque and relative peak strength

Mixed repeated-measures ANOVA revealed significant main effects of repetition (p<0.001) and a significant condition × repetition interaction (p<0.001; Table 1). Under severe BFR conditions, evoked torque declined progressively across repetitions. In the 50sev-BFR condition, torque at the fifth repetition was significantly lower than all preceding repetitions (first through fourth; p = 0.002), while the fourth repetition showed significant reductions compared to repetitions 1-3 (p = 0.016). In addition, the third repetition demonstrated decreased torque relative to the second repetition (p<0.001). The 75sev-BFR condition exhibited a similar but attenuated pattern, with the fifth repetition producing significantly less torque than the first, third, and fourth repetitions (p = 0.02), and the fourth repetition showing reduced torque compared to the third (p = 0.024). Between-condition comparisons revealed notable differences in torque generation capacity.

**Table 1:** Evoked torque and relative peak strength by repetitions and conditions Superscripts indicate significant post hoc pairwise comparisons: ^1, 2, 3, 4^versus the first, second, third, and fourth repetition, respectively; ^a^versus 50% maximum tolerable intensity combined with 50 mmHg applied pressure. Data are presented as mean ± standard deviation. Significant differences were set at p<0.05 (two-tailed). The general p-value corresponds to the overall ANOVA effect of repetition BFR: blood flow restriction; MTI: maximum tolerable intensity; NMES: neuromuscular electrical stimulation

Measure	NMES + BFR		Repetition					
	MTI	Pressure	First	Second	Third	Fourth	Fifth	P-value (overall ANOVA effect of repetition)
			Evoked torque (N • m)	50%	250 mmHg	96.72 ± 42.27^a^	96.77 ± 42.83^a^		89.53 ± 39.83^2^	82.60 ± 37.00^1,2,3^	74.94 ± 34.26^1,2,3,4^	<0.001
50 mmHg	81.41 ± 45.66	81.78 ± 44.31			80.50 ± 44.48	78.97 ± 43.16	77.69 ± 41.57					
0 mmHg	88.41 ± 43.51	90.01 ± 48.01			88.54 ± 46.27	88.26 ± 46.09^a^	87.47 ± 44.68^a^					
75%	250 mmHg	99.50 ± 36.25		95.07 ± 35.89	91.28 ± 30.59	84.14 ± 28.26^3^	76.44 ± 26.88^1,3,4^					
	50 mmHg	91.56 ± 33.13		89.97 ± 31.31	86.36 ± 33.40	83.87 ± 31.97	81.97 ± 33.35					
	0 mmHg	89.23 ± 32.24		88.87 ± 34.75	90.03 ± 34.55	88.83 ± 33.79	89.92 ± 33.42					
Relative peak strength (%)	50%	250 mmHg	77.80 ± 40.96^a^	77.44 ± 39.10^a^	71.47 ± 36.10^2^	66.45 ± 35.31^1,2,3^	60.75 ± 34.18^1,2,3,4^	0.001
		50 mmHg	66.92 ± 43.53	66.50 ± 43.02	65.52 ± 42.51	64.66 ± 42.31	63.10 ± 40.40	
		0 mmHg	71.83 ± 44.49	72.91 ± 45.94	72.32 ± 46.68^a^	71.55 ± 45.33^a^	70.96 ± 44.48^a^	
	75%	250 mmHg	89.05 ± 54.14	86.35 ± 55.71	81.95 ± 49.62	74.97 ± 44.38^1,3^	67.14 ± 37.54^1,3,4^	
		50 mmHg	83.64 ± 55.74	83.45 ± 58.44	81.02 ± 59.26	78.78 ± 58.14	77.61 ± 59.80	
		0 mmHg	80.73 ± 51.44	81.49 ± 55.28	82.63 ± 55.97	81.49 ± 55.65	82.63 ± 55.97	

During initial repetitions (first and second), the 50sev-BFR condition generated significantly higher torque than 50mild-BFR (p = 0.045). However, this advantage was no longer evident in later repetitions, where 50NMES produced significantly greater torque than 50mild-BFR at repetitions four and five (p = 0.027). Relative peak strength (torque normalized to MVC) exhibited parallel patterns. In 50sev-BFR, the fifth repetition showed significant decrements compared to all prior repetitions (p = 0.002), with the fourth repetition lower than repetitions one to three (p = 0.018), and the third lower than the second (p<0.001). The 75sev-BFR condition demonstrated reduced relative peak strength at the fifth repetition compared to the first, third, and fourth repetitions (p = 0.041), with the fourth repetition significantly lower than the first and third (p = 0.025). Between-condition analyses revealed that 50sev-BFR exceeded 50mild-BFR during early repetitions (first and second; p = 0.032), while 50NMES maintained higher relative peak strength than 50mild-BFR during later repetitions (third through fifth; p = 0.041).

Fatigue development

Fatigue analysis - quantified by the negative slope of torque decline across repetitions - revealed significant differences between conditions (Table 2). Severe BFR conditions produced markedly steeper fatigue slopes compared to all other conditions. Specifically, 50sev-BFR (slope: -5.77 ± 2.87) and 75sev-BFR (slope: -5.71 ± 6.61) demonstrated significantly greater fatigue than conditions without severe restriction (p = 0.013), with no significant difference between the two severe BFR conditions (p = 1.00).

**Table 2:** Fatigue determined via linear regression analysis for different experimental conditions ^*^Significantly different versus 50 mmHg and 0 mmHg conditions Data are presented as mean ± standard deviation. Significant differences were set at p<0.05 (two-tailed). The general p-value refers to the main effect of stimulation intensity in the repeated-measures ANOVA (p = 0.001) BFR: blood flow restriction; MTI: maximum tolerable intensity; NMES: neuromuscular electrical stimulation

Measure	NMES + BFR		Mean	95% confidence interval		P-value (one-way ANOVA)
	MTI	Pressure		Lower bound	Upper bound	
Fatigue slope	50%	250 mmHg	−5.77 ± 2.87^*^	−7.30	−4.24	0.001
		50 mmHg	−1.03 ± 2.72	−2.47	0.42	
		0 mmHg	−0.26 ± 3.02	−1.93	1.41	
	75%	250 mmHg	−5.71 ± 6.61^*^	−9.23	−2.18	
		50 mmHg	−2.53 ± 4.09	−4.71	−0.35	
		0 mmHg	0.13 ± 1.80	−0.82	1.09	

Pain perception

Pain scores demonstrated significant main effects of both BFR pressure and NMES intensity (Table 3). Severe BFR conditions elicited significantly greater pain compared to both mild restriction and unrestricted conditions (p<0.05). Additionally, a clear intensity-dependent response emerged, with 75% of maximum tolerable intensity producing significantly higher pain ratings than 50% intensity (p = 0.001). In addition, a significant BFR pressure × NMES intensity interaction was observed (p<0.05), indicating that the increase in pain perception with higher stimulation intensity was more pronounced under severe restriction compared to mild or unrestricted conditions. Post hoc comparisons revealed specific between-condition differences. Most notably, 50sev-BFR generated significantly greater pain than 50mild-BFR at the end of the protocol (p<0.05), demonstrating that even at moderate NMES intensities, severe vascular restriction substantially elevates discomfort.

**Table 3:** Pain scores observed under different experimental conditions Superscripts indicate significant post hoc pairwise comparisons. ^a^Versus 50% maximum tolerable intensity combined with 50 mmHg applied pressure Data are presented as mean ± standard deviation. Significant differences were set at p<0.05 (two-tailed). General p-values refer to the main effects of BFR pressure and NMES intensity in the repeated-measures ANOVA BFR: blood flow restriction; MTI: maximum tolerable intensity; NMES: neuromuscular electrical stimulation; VAS: visual analog scale

NMES + BFR		Pain (VAS scale)	95% confidence interval		P-value (ANOVA main effects)
MTI	Pressure		Lower bound	Upper bound	
50%	250 mmHg	5.48 ± 1.51^a^	4.67	6.28	0.001
	50 mmHg	4.68 ± 1.55	3.85	5.50	
	0 mmHg	4.78 ± 1.62	3.92	5.65	
75%	250 mmHg	6.54 ± 2.12	5.41	7.67	
	50 mmHg	6.13 ± 1.88	5.13	7.13	
	0 mmHg	5.98 ± 1.83	5.01	6.96	

## Discussion

Our investigation demonstrated that combining moderate-to-high intensity NMES with severe BFR pressure significantly accelerates muscle fatigue and elevates pain perception compared to isolated NMES or NMES with mild restriction. Specifically, our primary findings revealed that severe BFR conditions produced markedly steeper fatigue slopes independent of NMES intensity, with evoked torque declining substantially by the fourth and fifth repetitions. Intriguingly, the 50sev-BFR condition initially generated higher torque values than 50mild-BFR during early repetitions, suggesting an acute potentiation effect from severe blood flow restriction that dissipated as metabolic stress accumulated. In addition, pain perception demonstrated dose-dependent increases with both BFR pressure severity and NMES intensity, reaching maximum levels when 75% intensity was combined with severe restriction. Collectively, these findings indicate that while severe BFR may transiently enhance force production, the accelerated fatigue development and heightened discomfort may pose a potential barrier to clinical applicability, particularly in populations with reduced exercise tolerance. However, this remains to be confirmed in future studies directly involving such populations.

The mechanisms underlying BFR-induced acceleration of peripheral fatigue during NMES warrant careful examination. Our observations of increased fatigue under higher BFR pressures align with previous investigations [10,14] that documented torque reductions proportional to perfusion restriction severity. However, a striking difference emerged in the temporal pattern of fatigue development, with our protocol inducing significant decrements within merely five repetitions compared to 30-180 repetitions reported previously. This accelerated fatigue trajectory likely stems from fundamental differences in stimulation parameters, particularly our use of wider pulse widths (380 μs) and higher frequency (75 Hz) compared to the very short pulse widths and low frequencies employed in earlier studies [10,14]. Both wider pulse widths and higher frequency currents enhance motor unit recruitment and central torque development [3], producing effects comparable to those achieved through higher current intensities [15].

Despite the theoretical advantage of increased motor unit recruitment for sustaining force production, our results suggest that the metabolic consequences of high-frequency stimulation combined with vascular occlusion override any potential benefits. The critical role of stimulation frequency in metabolic demand and subsequent fatigue development has been elegantly demonstrated by Russ et al. [16], who investigated ATP consumption patterns in the medial gastrocnemius muscle under different frequencies. Their findings revealed that 80 Hz stimulation imposed significantly higher ATP costs per contraction and generated greater accumulation of inorganic phosphate with more pronounced pH changes compared to 20 Hz stimulation, establishing that higher pulse frequencies during NMES substantially increase peripheral fatigue through enhanced metabolic stress. Well-documented mechanisms of peripheral fatigue involve alterations at or distal to the neuromuscular junction driven by metabolic factors, including hydrogen ion accumulation, inorganic phosphate elevation, lactate production, and reactive oxygen species generation [17]. When severe BFR restricts clearance of these metabolites while simultaneously limiting oxygen delivery, the fatigue-inducing environment intensifies dramatically.

Although our protocol employed intermittent stimulation with 20-second rest periods between contractions, contrasting with the continuous protocols used by Cole and Brown [10] and Murthy et al. [14], the distinctions between continuous and intermittent electrical stimulation on fatigue development remain ambiguous [18]. Nevertheless, our results suggest that sustained isometric contractions elicited by high-frequency current under severe externally applied pressure conditions prove more fatiguing than intermittent contractions at very low frequency, even when using lower relative current intensities. The observation that 50sev-BFR generated higher initial torque values than 50mild-BFR before fatigue onset represents a particularly intriguing finding, suggesting that severe external pressure and consequent muscle hypoxia produce an acute reinforcement effect. This phenomenon likely results from increased excitatory drive to motor neurons under hypoxic conditions, manifesting as enhanced torque production [19]. The absence of this potentiation effect in the 75sev-BFR condition suggests that higher current intensity (75% versus 50% of maximum tolerable intensity) may already maximize motor unit activation [3], thereby obscuring any additional facilitation from BFR-induced neural adjustments. Further investigation is warranted to elucidate the precise mechanisms and potential clinical applications of this transient enhancement effect.

Pain perception during combined NMES and BFR represents a critical determinant of treatment feasibility; however, prior research offers limited understanding of the individual contributions of each modality. Our findings extend current understanding by demonstrating both independent and synergistic effects of NMES intensity and BFR pressure on subjective discomfort. While we observed main effects for both severe BFR and 75% maximum tolerable intensity, these results diverge from previous investigations [1,8] in important ways. Natsume et al. [1] measured pain following 23-minute sessions of combined NMES and BFR versus isolated NMES, reporting greater discomfort in the combined condition despite using very low current intensity (5-10% MVC). Given this minimal electrical stimulation, the externally applied pressure likely constituted the primary pain stimulus in their protocol.

More recently, Head et al. [8] employed maximum tolerable NMES intensity combined with three BFR pressures (40%, 60%, and 80% AOP), finding significant pain increases only at 80% AOP compared to NMES alone, with lower pressures showing no significant differences. Notably, they observed a trend toward increased pain perception with greater workload under BFR conditions. Our intermediate approach using submaximal intensities revealed that fewer repetitions might explain why we detected fewer condition-specific differences, though workload represents only one relevant variable. Critical comparison of pain scores between studies reveals that Natsume et al. [1] reported lower overall pain than Head et al. [8] at similar external pressures, with the primary distinction being NMES intensity (5-10% MVC versus maximum tolerable intensity). Our data showing greater pain interaction at 75% than 50% of maximum tolerable intensity (Table 3) confirms that NMES intensity may generate more pronounced discomfort than BFR-induced hypoxia.

The neurophysiological mechanisms generating pain during combined NMES and BFR involve distinct pathways that may interact synergistically. NMES-induced discomfort originates from electrical current penetrating cutaneous tissues [3] and activating both Aβ mechanoreceptors (type II sensory fibers) and, more significantly, Aδ and C afferent fibers (type III and IV sensory fibers) [20]. These nociceptive fibers transmit high-intensity NMES information perceived as pain or discomfort to central processing centers. The second pathway involves BFR-triggered physiological responses, where the severe metabolic environment created by exercise under restriction increases perception of exertion and pain compared to unrestricted conditions [21].

The hypoxic environment, combined with enhanced metabolite production and suppressed venous clearance, alters sensory feedback from group III-IV muscle afferents, increasing sympathetic nervous system activity and ultimately amplifying subjective pain perception [22]. While both pathways exhibit similar conduction velocities, their pain generation mechanisms differ fundamentally: NMES delivers instantaneous nociceptive input upon stimulation, whereas BFR-related discomfort develops progressively as metabolic stress accumulates with exercise volume rather than pressure alone, resulting in greater nociceptive information transmission as hypoxia intensifies.

Our specific finding that only 50sev-BFR provoked significantly higher discomfort than 50mild-BFR deserves particular attention. While high externally applied pressure is well-established to increase discomfort levels [7], lower pressure values (50 mmHg) may paradoxically induce analgesic effects through gate control mechanisms. According to gate control theory, large myelinated fibers (Aβ fibers) responding to pressure and vibration can activate spinal interneurons that modulate or inhibit secondary neurons linked to nociceptors [23], potentially explaining the reduced discomfort observed in the 50mild-BFR condition through partial inhibition of NMES-induced pain signals.

This study has several limitations requiring acknowledgment, including sample size constraints and inherent challenges of crossover designs. Although crossover methodology efficiently compares treatment effects, it carries risks of order effects and treatment-period interactions. In this study, we implemented the approach proposed by Cleophas [24] to assess and minimize bias risk through systematic data evaluation and individual period reporting. Our decision to employ static externally applied pressures rather than individualized AOP percentages ensured complete vascular occlusion in severe conditions, but may have produced variable relative restriction levels across participants, given anthropometric differences. Conversely, the study's major strengths include systematic repetition-by-repetition fatigue assessment under different pressure and intensity combinations and evaluation of multiple NMES intensities below maximum tolerable levels, providing clinically relevant data for protocol optimization.

The practical implications of our findings suggest that combining NMES with BFR demands careful consideration of enhanced training stimuli against reduced tolerability and adherence potential. While severe BFR combined with moderate-to-high NMES intensity theoretically maximizes adaptation stimuli, rapid fatigue onset and elevated pain levels could substantially compromise treatment adherence, particularly among clinical populations already challenged by discomfort tolerance or chronic pain conditions. The transient torque potentiation observed with severe BFR suggests potential applications for protocols emphasizing brief, high-quality contractions rather than sustained training volumes, potentially exploiting the enhancement window before fatigue accumulation.

From an ecological validity perspective, lower intensity protocols may produce meaningful chronic adaptations - perhaps of lesser magnitude - while remaining feasible for clinical implementation. The finding that 50% maximum tolerable intensity generated substantial torque with reduced discomfort suggests this level may represent an optimal starting point for clinical protocols, with progression guided by individual tolerance and response rather than predetermined intensity schemes. Future research should evaluate chronic effects of moderate-intensity NMES combined with submaximal BFR pressures (e.g., 80% AOP) while systematically assessing session discomfort to establish protocols balancing efficacy with adherence in clinical populations.

## Conclusions

We demonstrated that combining high BFR pressure with moderate-to-high NMES intensity fundamentally accelerates muscle fatigue compared to NMES alone, with performance decrements evident by the fourth repetition. In addition, pain perception increased proportionally with both BFR pressure severity and NMES intensity, reaching maximum levels under severe restriction conditions. Despite these challenges, our findings indicate that medium-to-high intensity electrical stimulation (50-75% of maximum tolerable intensity) provides robust neuromuscular activation without requiring maximum tolerable intensity, while the transient torque potentiation observed during initial repetitions under severe BFR warrants further investigation. For clinical implementation, we recommend initiating protocols with 50% maximum tolerable NMES intensity combined with moderate BFR pressure (50 mmHg) to optimize therapeutic benefits while maintaining patient tolerability. Progression to higher intensities should be individually guided based on patient response and adherence.
